# Risk of Bias Tool in Systematic Reviews/Meta-Analyses of Acupuncture in Chinese Journals

**DOI:** 10.1371/journal.pone.0028130

**Published:** 2011-12-09

**Authors:** Yali Liu, Shengping Yang, Junjie Dai, Yongteng Xu, Rui Zhang, Huaili Jiang, Xianxia Yan, Kehu Yang

**Affiliations:** 1 Evidence-Based Medicine Center, School of Basic Medical Sciences, Lanzhou University, Lanzhou, China; 2 Institute of Integrated Traditional Chinese and Western Medicine, Lanzhou University, Lanzhou, China; 3 The First Clinical Medical College of Lanzhou University, Lanzhou, China; University of Illinois-Chicago, United States of America

## Abstract

**Background:**

Use of a risk of bias (ROB) tool has been encouraged and advocated to reviewers writing systematic reviews (SRs) and meta-analyses (MAs). Selective outcome reporting and other sources of bias are included in the Cochrane ROB tool. It is important to know how this specific tool for assessing ROB has been applied since its release. Our objectives were to evaluate whether and to what extent the new Cochrane ROB tool has been used in Chinese journal papers of acupuncture.

**Methods:**

We searched CBM, TCM database, CJFD, CSJD, and the Wanfang Database from inception to March 2011. Two reviewers independently selected SRs that primarily focused on acupuncture and moxibustion, from which the data was extracted and analyzed.

**Results:**

A total of 836 SRs were identified from the search, of which, 105 were included and four are awaiting assessment. Thirty-six of the 105 SRs were published before release of the Cochrane ROB tool (up to 2009). Most used the Cochrane Handbook 4.2 or Jadad's scale for risk or quality assessment. From 2009 to March 2011 69 SRs were identified. While “risk of bias” was reported for approximately two-thirds of SRs, only two SRs mentioned use of a “risk of bias tool” in their assessment. Only 5.8% (4/69) of reviews reported information on all six domains which are involved in the ROB tool. A risk of bias graph/summary figure was provided in 2.9% (2/69) of reviews. Most SRs gave information about sequence generation, allocation concealment, blindness, and incomplete outcome data, however, few reviews (5.8%; 4/69) described selective reporting or other potential sources of bias.

**Conclusions:**

The Cochrane “risk of bias” tool has not been used in all SRs/MAs of acupuncture published in Chinese Journals after 2008. When the ROB tool was used, reporting of relevant information was often incomplete.

## Introduction

Assessment of internal validity, risk of bias, or methodological quality of studies included in systematic reviews (SRs) and meta-analyses (MAs) is a very important step in identifying limitations of individual studies. Randomized controlled trials (RCTs) are often included as the study type for SRs of interventions.

Since the 1980s, numerous tools involving scales and checklists have been developed for assessing the methodological quality of clinical trials [1], including the Cochrane Collaboration's “risk of bias” (ROB) tool which was published in 2008 [2]. It shows that “the ROB tool is composed of two parts, ‘description’ and ‘judgment’. For parallel group trials, it addresses six specific domains: sequence generation, allocation concealment, blinding, incomplete outcome data, selective outcome reporting, and other sources of bias. In these six domains, the judgments of ‘Yes’, ‘No’, or ‘Unclear’ indicates ‘low risk of bias’, ‘high risk of bias’, and ‘uncertain risk of bias’, respectively” [2]. As an essential guide to writing a Cochrane SRs, use of the ROB tool has been encouraged and advocated [2]. Moreover, authors are encouraged to use the latest version, which is currently Handbook 5.1.0 [updated March 2011].

The ROB tool continues to be recommended and disseminated by the Cochrane Collaboration and its sub-centers in different countries. In determining the effect of the ROB tool, it is very important to know how effectively it has been applied. Accordingly, we evaluate whether and to what extent the Cochrane ROB tool has been used in SRs of acupuncture published in Chinese journals.

## Methods

The protocol of this study was written in Chinese which has not published.

### Inclusion Criteria

SRs or MAs of acupuncture/acupressure and moxibustion published on Chinese journals. We included studies that described their methods and results in detail.

### Exclusion Criteria

SRs and MAs primarily focused on the other traditional Chinese medicine (TCM) (herbal medicine, massage, etc) rather than acupuncture.

### Search Strategy (Text S1)

Five databases (Chinese Biomedicine Literature Database (CBM), Traditional Chinese Medicine database (TCM database), Chinese Journal Full-text Database (CJFD), Chinese Scientific Journal Full-text Database (CSJD), and Wanfang Database) were systematically searched from inception to March 2011. The main search terms were as follows: “systematic review”, “meta-analysis”, “acupuncture”, “needling”, “ear acupuncture”, “ electroacupuncture”, “electro-acupuncture”, “acupuncture points”, “acupressure”, “moxibustion”, and “acupoint”.

### Screening

Two reviewers (Yongteng XU and Huaili JIANG) independently screened the title and abstract of each record. Full texts of potentially included articles were further assessed. Disagreements were resolved by discussion.

### Data Extraction and Analysis

Data about general characteristics and “risk of bias” were independently extracted by two reviewers (Junjie DAI and Rui ZHANG). Discrepancies were resolved through discussion or settled by the third principal investigator (Yali LIU). Since the ROB tool was first published in February 2008 (Cochrane handbook 5.0.0) [3], we assessed the use of the ROB tool only in those reviews published since 2009.

Data was extracted into a standardized form by trained extractors. The forms was composed of two parts: (1) General information: publication, type of included studies, funding etc, and (2) Information related to risk of bias: name and version of assessment tool, risk of bias graph/summary; randomization sequence, allocation concealment, blinding, incomplete outcome data, selective reporting, and other potential sources of bias etc. Each domain was assessed as ‘yes’ (described in papers), or “no” (not described in papers).

Data was summarized using descriptive statistics (frequency, percentage). Analysis was carried out with Excel (version Microsoft Excel 2007; http://office.microsoft.com/zh-cn/) and SPSS software (version 13.0; http://www.spss.com).

## Results

### Search

**Figure 1:**
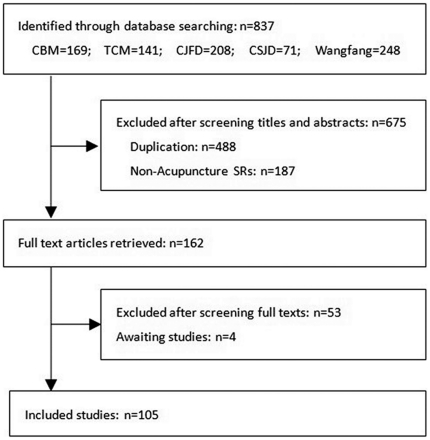
Flow chart of articles identified, included and excluded.

Our search identified 837 SRs and MAs, of which 675 abstracts did not meet inclusion criteria. One hundred and sixty-two reviews were chosen for full text analysis and assessed for inclusion. Full texts were obtained for 158 reviews; 105 met inclusion criteria and four are awaiting assessment, as full text was not available (Figure 1, Text S2). All SRs and MAs were written by Chinese authors.

### General Characteristics (Table 1)

**Table 1:** Characteristics of included studies.

Category	Characteristic	Number (%) ofn = 105
Title	Systematic review	74 (70.5)
	Meta analyse	22 (20.9)
	Others	9 (8.6)
Diagnostic criteria	Western medicine (diseases)	41 (39.0)
	Traditional medicine	2 (1.9)
	Using both disease and syndrome	15 (14.3)
	No diagnostic criteria reported	47 (44.8)
funding source	The number of reviews with funding source(s)	59 (56.2)
	Chinese foundation	58 (98.3*)
	International foundation	1 (1.7*)
	No declarations of interest	56 (94.9*)
	The number of funding sources	Median:1 (range: 0–5)
Trial types included	RCTs	96
	CCT	38
	quasi-RCTs	14

*n = 59.

The first systematic review and meta-analysis were published in 2002 and 2003, respectively. Since 2007, the number of SRs and MAs published annually has increased.

Of the 105 included reviews, 74 and 22 included “systematic review” and “meta-analysis” in their titles, respectively. However, there were nine reviews that included “evidence-based medicine analysis”, “the curative effect comparison appraises”, and other phrases in their titles, which were later identified as “systematic reviews” or “meta-analyses.” All reviews concerned diseases defined from a western medicine perspective. In total, 15 different types of diseases were involved, with the majority [24.8% (26/105)] focused on treatment of diseases of the nervous system. Diagnostic criteria were reported in 55.2% (58/105) of the reviews. Of these 58 reviews, 41 reported their diagnostic criteria based solely on “Western disease” and two reviews reported their diagnostic criteria based solely on “TCM syndrome.” The remaining 15 reviews included both “western disease” and “TCM syndrome” diagnostic criteria. Funding was supplied by at least one funding body for 56.2% (59/105) of reviews. Of these, 98.3% [58/59] were supported by funding from China and only one was funded by an international foundation (The China Medical Board, CMB). Most, 94.9% (56/59), failed to provide declarations of interest, while the remaining three reviews reported that there were no conflicts of interests.

While most [91.4% (96/105)] SRs and MAs restricted study design to RCTs, some included controlled clinical trials (CCTs) [36.2% (38/105)] and quasi-RCTs [13.3% (14/105)]. The number of trials included in the reviews ranged from three to 203, with a median of 12.7. And the number of included RCTs ranged from 0 to 67, with a median of 7.

### Risk of bias tool (Table 2, 3)

**Table 2:** The use of numerous tools in studies.

Assessment tools	2000–2008 y (n = 36)	2009–2011.3 y (n = 69)
Cochrane Handbook version 4	7	25
Cochrane Handbook version 5	-	13
Cochrane Handbook (version not reported)	5	13
Jadad scale	12 (11+1*)	40 (15+25*)
Juni	3	1
PED pro	1	0
No mention assessment tool	7	2 (1+1#)
Others	2	0

*The number of reviews that applied both the Jadad scale and another.

# No use any assessment tool.

**Table 3:** Reporting of the six domains in the ROB tool in SRs and MAs after 2009.

ROB tool's domains	Systematic reviews(n = 50)	Meta-analyses(n = 13)	Others titles(n = 6)	Subtotal(n = 69)
Sequence generation	48	13	6	67
Allocation concealment	42	9	5	56
Blindness	49	13	6	68
Blinding participants	1	1	0	2
Blinding healthcare providers	0	1	0	1
Blinding outcome assessors	2	0	0	2
Blinding data analysts	2	0	0	2
Incomplete outcome data	7	1	0	8
Loss of follow-up	43	7	6	56
Intention-to-treat (ITT) analysis	14	4	1	19
Selective outcome reporting.	4	0	0	4
Other potential sources of bias	4	0	0	4
Using ROB graph/summary	2	0	0	2
Reported verbatim quotes	0	0	0	0

Thirty-six SRs were published during the seven years from 2002 through 2008, and another 69 since 2009. Of the first 36 SRs, one-third [33.3% (12/36)] used the Jadad scale [4] and one-third [33.3% (12/36)] applied the Cochrane Handbook. Among the latter 12 reviews, seven used the Cochrane Handbook 4, and another five reviews used the Cochrane handbook but failed to report the exact version used. Most reviews assessed sequence generation, allocation concealment, blinding, loss of follow-up and intention-to-treat (ITT) analysis.

Of the 69 SRs published since 2009, 73.91% (51/69) applied the Cochrane Handbook as an assessment tool. Of these, 18.84% (13/69) used the Cochrane Handbook 5 and, 36.23% (25/69) used the Cochrane Handbook 4, and a further thirteen reviews used the Cochrane handbook but failed to report the version used. The Jadad scales were used by 57.97% (40/69) and of these, 25 used both the Cochrane Handbook and the Jadad scale. One review did not use any quality or ROB assessment for included studies. Methodological quality and ROB have been used interchangeably in the SRs and MAs. Most reviews used “quality assessment” rather than “risk of bias assessment” in their methods or results. Only two reviews specified a “risk of bias tool” as their assessment tool.

Few [5.8% (4/69)] reviews reported on all six domains of the Cochrane ROB tool. Most SRs gave information about baseline similarity; however, only four reviews described other potential sources of bias and selective reporting bias. A “risk of bias graph/summary” figure was provided in 2.9% (2/69) of the reviews.

Information about blinding was reported in 68 reviews, but 61 of these failed to report who was blinded in the trials. Most reviews reported loss of follow-up or ITT analysis, but failed to mention incomplete outcome data. None of the reviews reported verbatim quotes in their papers.

## Discussion

An increasing number of SRs and MAs of acupuncture have been published, especially since 2007. In this study, we identified 105 SRs and MAs of acupuncture interventions in Chinese journals. In addition, there were 36 Cochrane SRs in the Cochrane Database of Systematic Reviews (CDSR) and 154 SRs and MAs have been published in international journals that primarily focus on acupuncture. To our knowledge, this paper is the first to investigate the use of the Cochrane Collaboration's ROB tool in the acupuncture field. Although the study was not a classical systematic review, we tried to report it according to PRISMA Checklist [14] (Text S3).

We identified other studies that focused on use of the ROB tool. While most of these studies evaluated the ROB of RCTs and/or their influence in specific fields, such as dentistry [5], pediatrics [6], and persistent asthma treatment [7], other studies have assessed the internal validity of RCTs, inter-rater agreement [8], [9], and concurrent validity [8], [9]. Some reviews have contrasted the ROB tool with other tools, such as the Jadad scale [7], [8], the Schulz approach [7], [8], and the Effective Public Health Practice Project Quality Assessment Tool (EPHPP) [9]. Hartling et al. demonstrated low correlation and varied inter-rater agreements between the ROB tool assessments and the Jadad scale [7], [8].

Other reviews of SRs and MAs published in Chinese journals have identified problems with methodological or reporting quality, however, these studies failed to pay attention to use of the ROB tool in their reviews [10]–[12].

The QUOROM statement [13] and the updated version of the PRISMA statement [14] encourage use of the terms systematic review or meta-analysis in titles of such studies, in order to maximize search success. Among the reviews we identified, most included these terms in their titles, however, nine SRs or MAs failed to use these terms in their titles in Chinese. In addition, most reviews did not declare whether a conflict of interest existed or not, which is a potential threat to validity.

Although acupuncture belongs to the domain of TCM, most SRs and MAs focus on “western disease” rather than “TCM syndrome” as their diagnostic criteria. Consequently, TCM practitioners may find it difficult to understand and apply evidence from such publications in their clinical practice because TCM places more emphasis on syndromes rather than on western disease classifications. Therefore, some researchers propose using both western disease and TCM syndrome in their diagnostic criteria in studies of TCM. This is an interesting issue worthy of serious discussion but it is beyond the scope of this paper.

Many instruments exist for assessing the “quality” of RCTs. While 25 scales and nine checklists were published prior to 1994 [15], [16], more instruments focusing on methodological quality [2], [4], [17]–[20] or both methodological quality and reporting quality [21]–[24] have since been proposed. Despite the abundance of such instruments, assessment tools specifically for evaluating acupuncture RCTs are rare [23]. In addition, some items of these instruments are not directly related to internal validity. For example, whether a power calculation was done or not relates to the precision of the results rather than internal validity [2]. In our study, we concentrated on tools that focused on internal validity.

We found that the Cochrane Handbook and the Jadad scale were often chosen by Chinese reviewers. Although, the Jadad scale has been popular for many years, using the Cochrane Collaboration ROB tool instead of the Jadad scale should be recommended when performing SRs and MAs. There are several core reasons for this recommendation: 1) the Jadad scale is based on reporting quality rather than actual RCT conduct [4]; 2) while the Jadad scale focuses on randomization, double blinding, withdrawals or dropouts, we believe these criteria are insufficient to assess the internal validity of RCTs; 3) the Jadad scale tends to overestimate treatment effects because it ignores allocation concealment [25] and selective outcome reporting [26], [27], which are very important to overall assessment of ROB; and 4) the term “double blind” is incomplete and lacks specificity for assessing “blinding” because it is not clear who is blinded [28].

We found that most Chinese SRs and MAs were written according to the Cochrane Collaboration's Handbook. The Cochrane Handbook is frequently updated, the most recent being version 5.1.0, published in March 2011 [2]. Although many scales and tools are available for assessing methodological quality of RCTs, the new version of the Cochrane handbook recommends that a specific ROB tool be used assess the risk of bias in each included study. It is not uncommon that authors of SRs and MAs use the terms “methodological quality” and “risk of bias” interchangeably. The term “bias” indicates a systematic error or deviation from the truth in a study's results or inferences [2]. Assessing ROB is to directly assess the extent that the results of included studies should be *believed* [2]. But not all parts of quality assessment have direct implications for ROB. Therefore, ROB is recommended for assessing “bias” instead of methodological quality, because “bias” may be different from “quality”. For example, blinding is difficult or impossible for some interventions, such as surgery or Chinese herbal medicine. In these cases, the risk from lack of blinding may affect the trial's validity, however, it may be inappropriate to score these studies as “low quality” [2].

The Cochrane ROB tool was recommended in Cochrane handbook version 5.0.1, although ‘selective reporting’ and ‘other potential sources of bias’ were mentioned in Handbook 4.2.6. Few of the reviews we identified [18.8% (13/69)] reported that they used Cochrane Handbook 5 and only three of these also used all 6 domains. One review reported use of Cochrane Handbook 4.2.6 and also described the selective outcome reporting bias and the other potential sources of bias. Most SRs and MAs reported information about sequence generation, allocation concealment, blindness, and incomplete outcome data, however, major reviews ignored selective outcome reporting and other potential sources of bias. These studies that lack analysis of selective outcome reporting and other sources of bias have performed incomplete ROB assessment. Although baseline imbalance was described, many SRs and MAs failed to analyze the influence of other sources of bias. None of the SRs and MAs in our study analyzed early stoppage, conflict of interest, or other factors that are other potential threats to validity. Some of the SRs and MAs that reported using Cochrane Handbook 5 in their methods, actually used version 4 in their results. It is easy to mislead a reader because most clinicians may not know the differences between Cochrane Handbook 4 and Handbook 5.

With regards to blinding, we noticed that most reviews failed to describe the blinding process in detail. In a clinical trial, different types of personnel can be blinded, such as participants, healthcare providers, outcome assessors, and data analysts. If we do not know which types of personnel were blinded, it is difficult to accurately judge which bias (performance bias or measurement bias) may have occurred. In addition, use of the term “double blind” is ambiguous and authors often fail to state exactly who was blinded [28]. Some people assume “double blind” means that patients and clinicians were blinded, however, some authors reported “double blind”, when patients and outcome assessors were blinded. Furthermore, for subjective outcomes, blinding outcome assessors is more important than blinding clinicians in order to avoid measurement bias.

While most reviews provided details of loss to follow-up (attrition/drop-out) and ITT analyses, few mentioned the term “incomplete outcome data”. We recommend that future reviews include information about incomplete outcome data, not only the amount and distribution of drop-outs across study groups but also the reasons for outcomes being absent. This would help reviewers assess the risk of attrition bias.

The “risk of bias summary” figure was provided in few reviews. Reasons for this may be that reviewers are not be aware of this requirement or that journals may require specific layout specifications that authors are unable to comply with.

In order to improve assessment of risk of bias, we recommend that the most recent version of the Cochrane ROB tool be used by SR and MA authors. Reviewers should continue to update their knowledge according to the latest Cochrane Collaboration Handbook versions and other developing methodology and to clearly state which version of the tool or handbook was used in their reviews.

There are several limitations in the study. We included SRs and MAs that primarily focused on acupuncture. Those reviews involving acupuncture as a secondary intervention were excluded. We only selected SRs and MAs published in Chinese journals and therefore our results are only applicable to those journals. In addition, we did not analyze internal validity and inter-rater agreements between the ROB tool assessments and the Jadad scale or other assessment tools.

In conclusion, the Cochrane Handbook and the Jadad scale were the risk of bias or quality assessment instruments most commonly used by Chinese authors of systematic reviews and meta-analyses of acupuncture. In reviews published after 2008 in Chinese journals, Cochrane ROB tools were not always used. In cases where a Cochrane ROB tool was used, reporting was sometimes incomplete.

## Supporting Information

Text S1 **Five Chinese databases search strategy.**
(DOC)

Text S2 **One hundred and five SRs/MAs of acupuncture published in Chinese journals.**
(DOC)

Text S3 **PRISMA Checklist.**
(DOC)
