# Supplementary material for: Risk of Bias Tool in Systematic Reviews/Meta-Analyses of Acupuncture in Chinese Journals
Source: PLoS One. 2011 Dec 9;6(12):e28130. doi: 10.1371/journal.pone.0028130 (PMC3235108; doi:10.1371/journal.pone.0028130)
Supplement: Text S1 — Five Chinese databases search strategy. (DOC) [file pone.0028130.s001.doc]

**Text S1 Five Chinese databases search strategy.**

**Chinese Biomedicine Literature Database (CBM) search strategy:**

***Hyperlink address：***<http://sinomed.imicams.ac.cn/index.jsp>

#1 Systematic review/exp

#2 Systematic review

#3 Meta analysis/exp

#4 Meta analysis

#5 #1 or #2 or #3 or #4

#6 acupuncture/exp

#7 Acupuncture Therapy/exp

#8 Acupuncture Points/exp

#9 Moxibustion/exp

#10 acupuncture

#11 moxibustion

#12 acupressure

#13 acupoint

#14 acupuncture point

#15 electroacupuncture

#16 auricular acupuncture

#17 ear Acupuncture

#18 #6 or #7 or #8 or #9 or #10 or #11 or #12 or #13 or #14 or #15 or #16 or #17

#19 #5 and #18

**Traditional Chinese Medicine database (TCM database)**

***Hyperlink address:*** http://cowork.cintcm.com/engine/windex.jsp

#1 Meta analysis/exp

#2 Meta analysis

#3 Systematic review

#4 #1 or #2 or #3

#5 acupuncture/exp

#6 Acupuncture Therapy/exp

#7 Acupuncture Points/exp

#8 Moxibustion/exp

#9 acupuncture

#10 moxibustion

#11 acupressure

#12 acupoint

#13 acupuncture point

#14 Electroacupuncture

#15 auricular acupuncture

#16 ear Acupuncture

#17 #5 or #6 or #7 or #8 or #9 or #10 or #11 or #12 or #13 or #14 or #15 or #16

#18 #4 and #17

**Chinese Scientific Journal Full-text Database (CSJD) search strategy**

***Hyperlink address：***<http://www.cnki.net/>

#1 Systematic review

#2 Meta analysis

#3 #1 or #2

#4 acupuncture

#5 moxibustion

#6 acupressure

#7 acupoint

#8 acupuncture point

#9 Electroacupuncture

#10 auricular acupuncture

#11 ear Acupuncture

#12 #4 or #5 or #6 or #7 or #8 or #9 or #10 or #11

#13 #3 and #12

**Chinese Journal Full-text Database (CJFD) search strategy**

***Hyperlink address：***<http://www.cqvip.com/>

#1 Systematic review

#2 Meta analysis

#3 #1 or #2

#4 acupuncture

#5 moxibustion

#6 acupressure

#7 acupoint

#8 acupuncture point

#9 Electroacupuncture

#10 auricular acupuncture

#11 ear Acupuncture

#12 #4 or #5 or #6 or #7 or #8 or #9 or #10 or #11

#13 #3 and #12

**Wanfang Database search strategy**

Hyperlink address：<http://www.wanfangdata.com.cn/>

#1 Systematic review

#2 Meta analysis

#3 #1 or #2

#4 acupuncture

#5 moxibustion

#6 acupressure

#7 acupoint

#8 acupuncture point

#9 Electroacupuncture

#10 auricular acupuncture

#11 ear Acupuncture

#12 #4 or #5 or #6 or #7 or #8 or #9 or #10 or #11

#13 #3 and #12
