# Supplementary material for: Risk of Bias Tool in Systematic Reviews/Meta-Analyses of Acupuncture in Chinese Journals
Source: PLoS One. 2011 Dec 9;6(12):e28130. doi: 10.1371/journal.pone.0028130 (PMC3235108; doi:10.1371/journal.pone.0028130)
Supplement: Text S2 — One hundred and five SRs/MAs of acupuncture published in Chinese journals. (DOC) [file pone.0028130.s002.doc]

***Text S2 One hundred and five SRs and MAs of acupuncture published in Chinese journals.***

1. Li N, Feng B, Zou J, Liu Y (2002) Meta-analysis of acupuncture for semiplegia caused by stroke. Journal of Chengdu University of Traditional Chinese Medicine 25:37-39.
2. Wu B, Wen CY, Shi JL ,Li N, He J (2003) A meta-analysis of Acupuncture for treatment of drug addiction. Chinese Acupuncture & Moxibustion 23: 501-505.
3. Peng WN, Zhao H, Liu ZS, Liu BY (2004) Systematic review of electroacupuncture treatment for vascular dementia. Chinese Acupuncture & Moxibustion 24:297-301.
4. Li H, liang WX, Guo XF (2004) Meta-analysis of consciousness-restoring and orifice-opening acupuncture manipulation in treating stroke. Journal of Guangzhou University of Traditional Chinese Medicine 21:215-219.
5. Zhang SH, Liu M, Li L (2005) Acupuncture in the treatment of acute stroke: a Cochrane systematic review of randomized or quasi-randomized controlled trials. Chinese Journal of Clinical Rehabilitation 9:108-110.
6. Li Y, Luo C (2005) Acupuncture for tension-type headache: A systematic review. Chinese Journal of Evidence-Based Medicine 5:117-124.
7. Yu JN, Liu BY, Liu ZS, Chen XY (2005) Effectiveness and safety of acupuncture for mammary dysplasia. Chinese Journal of Evidence-Based Medicine 5:381-385,403.
8. He L, Zhou MK, Zhou D, Li N, Wu B (2005) Acupuncture for bell’s palsy: A systematic review. Chinese Journal of Evidence-Based Medicine 5:106-109,129.
9. Li N, Wu B, Wang CW, Kang J, Li HG (2005) A systematic review of randomized controlled trials for acupuncture and moxibustion treatment of insomnia. Chinese Acupuncture & Moxibustion 25:7-10.
10. Luo S, Luo SW (2005) Systematic review of acupuncture for treating intervertebral disk displacement. Journal of Clinical Acupuncture and Moxibustion 24:10-14.
11. Yang G, Wang XJ (2006) Electro-acupuncture for cervical spondylosis: A systematic review. Beijing Journal of Traditional Chinese Medicine 25:433-435.
12. Zhang W, Liu ZS, Peng WN (2006) A Systematic review of acupuncture therapy for benign prostatic hypertrophy. The Journal of Evidence-Based Medicine 6:291-296.
13. Wang LP, Xie Y (2006) Systematic review on acupuncture and moxibustion for treatment of dysphagia after stroke. Chinese Acupuncture & Moxibustion 26:141-146.
14. Liao L, Wei QP (2007) Evidence-based medicine analysis of academic theses in China of acupuncture therapy in optic atrophy treatment. Journal of Traditional Chinese Ophthalmology 17:208-210.
15. Liu XQ, Deng JF, Lin DK (2007) The evaluation of clinical articles about cervical spondylotic rdiculapthy treated with ACUPOTOMY. Chinese Journal of Traditional Medical Traumatology & Orthopedics 15:34-37.
16. Yang ZX, Shi XM (2007) Systematic review of the therapeutic effect and safety of Xingnao Kaiqiao needling method in treatment of stroke. Chinese Acuponcture & Moxibustion 27:601-608.
17. Zhang BY, Niu JM, Wen JY (2007) Meta-analysis of acupuncture for delivery. Shandong Journal of Traditional Chinese Medicine 26:547-549.
18. Yu XM, Zhu GM, Chen YL, Fang M, Chen YN (2007) Systematic review of acupuncture for treatment of herpes zoster in domestic clinical studies. Chinese Acupuncture & Moxibustion 27: 536-540.
19. Ma TM, Bai ZH, Ren L, Liu XL (2007) Meta analysis on the effect of acupuncture treatment on anxiety. Chinese Journal of Information on Traditional Chinese Medicine 14:101-103.
20. Gong XM, Ren K (2007) Acupuncture therapy and rehabilitation therapy for ankylosing spondylitis: A systematic review. Chinese Journal of Rehabilitation Medicine 22: 537-539.
21. Zhao T, Wang YH (2007) Systematic review of randomized controlled trials of acupuncture-moxibustion treatment of herpes zoster. Shanghai Journal of Acupuncture and Moxibustion 26:30-33.
22. Peng WN, Wang Y, Liu BY, Liu ZS, Mao M (2007) Systematic review of acupuncture for frozen shoulder. World Journal of Acupuncture-Moxibustion 17: 1-15.
23. Mu JP, Wu HG, Zhang ZQ, Liu HR, Zhu Y (2007) Meta analysis on acupuncture and moxibustion for treatment of ulcerative colitis. Chinese Acupuncture & Moxibustion 27:687-690.
24. Wang F, Zhang T, Liu Y (2007) Acupuncture for sciatica : A systematic review. World Chinese Medicine 2:354-355.
25. Song HM, Chen SQ, Wang SZ, Wu TX (2008) Effects of conservative treatment on cervical myelopathy: A systematic review. Journal of Clinical Rehabilitative Tissue Engineering Research 12:9149-9152.
26. Xu XD, Wu YJ (2008) Meta-analysis of effects of electric acupuncture on depression. Journal of Clinical Psychiatry 18:111-112.
27. Li QY, Peng WN, Mu Y, Xu YZ, Jiang YB, et al (2008) The systematic review of electroacupuncture for lumbar intervertebral disc herniation. Modern Journal of Integrated Traditional Chinese and Western Medicine 17:325-330.
28. Wang L, Chi QB (2008) Electro-acupuncture for depression: A systematic review of randomized controlled trials. Shanghai Journal of Acupuncture and Moxibustion 27:36-38.
29. Yang SL, Chen LD, Tao J, Zhang B (2008) Meta-analysis of randomized controlled trials on acupuncture and moxibustion treating depression. Chinese Journal of Rehabilitation Medicine 23:649-652.
30. Sun YL, Chen SB, Gao Y, Xiong J (2008) Acupuncture versus western medicine for depression in China: A systematic review. Chinese Journal of Evidence-Based Medicine 8:340-345.
31. Wang L, Sun DW, Zou W, Zhang JY (2008) Systematic review of therapeutic effect and safety of acupuncture for treatment of depression. Chinese Acupuncture & Moxibustion 28:381-386.
32. Guo XX, Jin HS, Huo L, Zheng J, Zhou XM (2008) Meta-analysis on acupuncture for treatment of dementia. Chinese Acupuncture & Moxibustion 28:140-144.
33. Yi K, Wang YF, Tian JH, Yang KH, Ma B, et al (2008). Acupuncture for rheumatoid arthritis：A systematic reviews. Gansu Science and Technology 24:144-146,162.
34. Wang CY, Han RF (2008) Acupuncture for chronic prostatitis: A meta-analysis. National Journal of Andrology 14:853-856.
35. Zhong BL, Huang YY, Li HJ (2008) The effectiveness and safety of acupuncture for depression: A systematic review. Chinese Mental Health Journal 22:641-647.
36. Liu ZD, Li HY, Song Y, Zhang BH, Guo JW, et al (2008) A systematic review of acupuncture-moxibustion treatment for stroke-induced motor dysfunction on the literature on clinical randomized controlled trials. Shanghai Journal of Acupuncture and Moxibustion 127:38-42.
37. Jiang GH, Xu XY, Chen ZH, Zhou ZH, Xu ZQ (2009) Systematic review of the therapeutic effect of CT-aided enchosure needling method in treatment of stroke. Journal of Clinical Acupuncture and Moxibustion 25:13-15.
38. Zhu MJ, Zhang H (2009) Meta-analysis in treating vascular dementia with different acupuncture methods. Liaoning Journal of Traditional Chinese Medicine 36: 1475-1477.
39. Lin HL, Song HM, Zhong WH, Chen SQ, Wang SZ (2009) Effects of different acupuncture and moxibustion treatments on cervical spondylotic radiculopathy: A systematic evaluation. Journal of Clinical Rehabilitative Tissue Engineering Research 13: 9017-9021.
40. Wang JH, Chen HP, Chen J (2009) Systematic review of randomized controlled trial on treating herpes zoster by fire needle. Journal of Clinical Acupuncture and Moxibustion 25: 16-18.
41. Fu L, Gao C (2009) Meta analysis of clinical randomized controlled trial about jiaji points on herpes zoster. Chinese Journal of Dermatovenereology of Integrated Traditional and Western Medicine 8: 112-117.
42. Li B, Xiong J, Du YH, Chai H, Lin XM (2009) Comparison of therapeutic effects between routine acupuncture and penetration needling on peripheral facial paralysis: A systematic review. Liaoning Journal of Traditional Chinese Medicine 36: 1381-1383.
43. He J, Zheng M, He CQ, Lan Q, Qu Y, et al (2009) Systematic review of acupoint stimulating therapy for deglutition disorders after apoplexy. Chinese Acupuncture & Moxibustion 29:66-71.
44. Luo XF, Li B, Du YH, Xiong J, Shi L (2009) Curative evaluation of peripheral facial paralysis by acupuncture combined with acupoint and intramuscular injection in controlled and randomized trials. Tianjin Journal of Traditional Chinese Medicine 26:27-29.
45. Sun P，Du Y，Xiong J，Li B (2009) Acupuncture versus traction for cervical spondylotic radiculopathy: A systematic review. Guangming Journal of Chinese Medicine 24:1824-1830.
46. Yue SJ, Fu LX, Lu YM, Wang ZL, Qi YZ (2009) Systematic review of therapeutic effect of acupuncture and medicine for treatment of generalized anxiety disorder. Journal of Clinical Acupuncture and Moxibustion 25:42-44.
47. Liu Q, Yu B, Du YH, Xiong J, Lin XM (2009) The curative effect comparison appraises of acupuncture near and far acupoints on acute lumbar muscle sprain. Liaoning Journal of Traditional Chinese Medicine 36:1392-1394.
48. Lin XM, Li B, Du YH, Xiong J, Sun P (2009) Systematic review of therapeutic effect of acupuncture for treatment of simple obesity. Chinese Acupuncture & Moxibustion 29:856-860.
49. Zhang T, Zhang L, Zhang HM, Li Q (2009) Systematic review of acupuncture therapy for acute ischemic stroke. China Journal of Traditional Chinese Medicine and Pharmacy 24:101-104.
50. Zhang W, Peng WN, Liu ZS (2009) Acupuncture for chronic fatigue syndrome: A systematic review. Journal of Evidence-Based Medicine 9:41-47.
51. Yu XM, Na MH, Chen YL (2009) A systematic review of acupuncture therapy for chronic non-bacterial prostatitis. Acta Universitatis Traditionis Medicalis Sinensis Pharmacologiaeque Shanghai 23:47-49.
52. Qi WZ, Fu LX, Xiong J, Wang ZL, Mou J, et al (2009) Systematic review of acupuncture for treatment of post-stroke spastic paralysis. Chinese Acupuncture & Moxibustion 29:683-688.
53. Zhang JB, Ren L, Sun Y (2009) Meta-analysis on acupuncture for treatment of depression in patients of poststroke. Chinese Acupuncture & Moxibustion 29:599-602.
54. Chai H, Li B, Du YH (2009) Effect analysis for acupuncture and moxibustion treatment of knee osteoarthritis. Liaoning Journal of Traditional Chinese Medicine 36:1197-1200.
55. Xiong J, Du YH, Liu JL, Lin XM, Sun P, et al (2009) Acupuncture versus western medicine for depression neurosis: A systematic review. Chinese Journal of Evidence-Based Medicine 9:969-975.
56. Xiao L, Li B, Du YH, Xiong J, Gao X (2009) Systematic review of the randomized controlled trials about acupuncture and moxibustion treatment of allergic rhinitis. Chinese Acupuncture & Moxibustion 29:512-516.
57. Li B, Chai H, Du YH, Xiao L, Xiong J (2009) Evaluation of therapeutic effect and safety for clinical randomized and controlled trials of treatment of acne with acupuncture and moxibustion. Chinese Acupuncture & Moxibustion 29:247-251.
58. Jin LW, Zhu J (2009) Acupuncture for hypotension: A systematic review. Journal of Clinical Acupuncture and Moxibustion 25:37-41.
59. Luo SY, Yang J, Chen MG (2009) A Meta-analysis of acupuncture in rheumatoid arthritis. Chinese Journal of Health Statistics 26:431-433.
60. Wang JJ, Song YJ, Wu ZC, Chu XO, Wang XH, et al (2009) A meta-analysis on randomized controlled trials of acupuncture treatment of chronic fatigue syndrome. Acupuncture Research 34:421-428.
61. LiB, Shi L, Du YH, Xiong J, Chai H (2009) Systematic review on randomized controlled trials of acupuncture therapy for chronic urticaria. Journal of Traditional Chinese Medicine 50:432-436.
62. Wang YN, Li B, Du YH, Li LN, Liu JL, et al (2009) Effectiveness and safety of randomized controlled trials of acupuncture of in the treatment of neurodermatitis. Liaoning Journal of Traditional Chinese Medicine 36: 2160-2163.
63. Liu ML, Lan L, Tang Y, Liang FR (2009) Acupuncture and moxibustion for breech presentation: A systematic review. Chinese Journal of Evidence-Based Medicine 9: 840-843.
64. Lan L, Liu ML, Tang Y, Liang FR (2009) Follow-up evaluation of therapeutic effects on acupuncture in primary dysmenorrhea. Journal of Shandong University of Traditional Chinese Medicine 33:511-514.
65. Lu YM, Fu LX, Mu, Xu HJ, Qi YZ (2009) Acupuncture for post stroke shoulder hand syndrome: A systematic review. Chinese Journal of Evidence-Based Medicine 9:976-978.
66. Chen W, Ma WP, Yu HH, Liu BW, Tu YM, et al (2009) Meta-analysis on acupuncture and moxibustion for treatment of vertebrobasilar insuffciency. Chinese Journal of Information on Traditional Chinese Medicine 16:107-112.
67. Li L, Zhan HS, Gao NY, Chen B, Shi YY (2010) Clinical randomized controlled trials on treatment of lumbar disc herniation by electro-acupuncture. China Journal of Traditional Chinese Medicine and Pharmacy 25:1949-1952.
68. Zhao Q, Cai BJ (2010) Acupuncture and massage treating cervical spondylosis of vertebral artery type in China: A systematic review. Journal of Changchun University of Traditional Chinese Medicine 26:217-218.
69. Huang XD, Fu LX, Li S, Wang ZL, Zhao R (2010) Systematic review of therapeutic effect of acupuncture combined with auricular point sticking and acupuncture for treatment of insomnia in clinical study. Journal of Clinical Acupuncture and Moxibustion 26:56-59.
70. Cai Y, Peng CX (2010) Meta-analysis on acupuncture treatment of diabetes. Chinese Archives of Traditional Chinese Medicine 28:2412-2415.
71. Wang J, Wang QH, Wu JY (2010) Meta-analysis of acupuncture treatment for primary trigeminal neuralgia. China Journal of Traditional Chinese Medicine and Pharmacy 25:2003-2006.
72. Huang JJ, Peng WQ, Lei LM, Lai YM (2009) Systematic review of treating lumbar interveterbral disc protrusion by combined therapy of Chinese and western medicines. Information on Traditional Chinese Medicine 26:65-69.
73. Wang YW, Fu WB, Peng HG, Ou AH (2011) Systematic reviews of clinically tandomized controlled trials on warming acupunctures treating cervical spodylosis. Liaoning Journal of Traditional Chinese Medicine 38:340-344.
74. Xu HR, Liu ZS, Zhao H (2011) Systematic review for treatment with acupuncture for overactive bladder syndrome. Modern Journal of Integrated Traditional Chinese and Western Medicine 20:393-399.
75. Li S, FU LX, Huang XD, Zhao R (2011) Systematic review of acupuncture for treatment of pseudobulbar palsy due to wind stroke in clinical studies. Journal of Clinical Acupuncture and Moxibustion 27:1-6.
76. Cao P, Yang RD (2011) Acupuncture for diabetic peripheral neuropathy: meta-analysis. Guiding Journal of Traditional Chinese Medicine and Pharmacy 17: 97-101.
77. Zhao MH, Huang XD, Xiong J, Qi YZ, Li S (2009) Systematic review on therapeutic effect of acupuncture for allergic rhinitis. Chinese Journal of Otorhinolaryngology in Integrative Medicine 17:309-312.
78. Zhu D, Lv HW (2010) Effectiveness of P6 stimulation on postoperative nausea and vomiting: A meta-analysis. Chinese Journal of Evidence-Based Medicine 10:923-931.
79. Zhao Q, Feng W, Cai BJ (2010) Warming needle for osteoarthrosis of knee in China: A systematic review. Journal of Liaoning University of Traditional Chinese Medicine 12:49-51.
80. Lin XM, Du YH, Xiong J, Chen YW, Xiao L, et al (2010) Acupuncture versus western medicine for singultus after stroke in China: A systematic review. Chinese Journal of Rehabilitation Medicine 25:353-356.
81. Gao X, Du YH, Xiao L, Lin XM, Chen YW, et al (2010) Acupuncture versus western medicine for trigeminal neuralgia in China: A Systematic Review. Jiangsu Journal of Traditional Chinese Medicine 42: 52-54.
82. Huang Y, Yang C, Tian Y, Yao DM, Li WJ, et al (2010) Systematic review of small needle-knife therapy for lumbar intervertebral disc protrusion. Lishizhen Medicine and Materia Medica Research 21:2420-2422.
83. Li LN, Li B, Xiong J, Du YH (2010) A systematic review of randomized controlled trials for acupuncture treatment of acute Bell's facial paralysis. Journal of Liaoning University of Traditional Chinese Medicine 12:97-99.
84. Pu HH, Yu T, Gao X, Mao JJ (2010) Systematic review on clinical therapeutic effect of acupuncture for treatment of gastrointestinal untoward reaction by malignant tumor chemotherapy. Lishizhen Medicine and Materia Medica Research 21: 1476-1480.
85. Gao, Du YH, Li B, Xiong J, Sun P, et al (2010) Acupuncture versus western medicine for fibromyalgia syndrome: A systematic review. Chinese Journal of Pain Medicine 16:112-114.
86. Tang HL, Pang J, Gao LF, Yang Y, Lei LM, et al (2010) Systematic review on treatment of sub-health with acupuncture-moxibustion and tuina in clinical researches. [Chinese Acupuncture & Moxibustion](http://acad.cnki.net/kns55/oldNavi/Bridge.aspx?LinkType=BaseLink&DBCode=cjfd&TableName=cjfdbaseinfo&Field=BaseID&Value=ZGZE&NaviLink=中国针灸) 30:699-703.
87. Li Y, Xiong J, Du YH, Li B, Shi L (2010) Acupuncture versus medicine for chronic prostatitis: A systematic review. Liaoning Journal of Traditional Chinese Medicine 37:1567-1572.
88. Peng H, Peng HD, Xu L, Lao LX (2010) Efficacy of acupuncture in treatment of cancer pain:a systematic review. Guiding Journal of Traditional Chinese Medicine and Pharmacy 8: 501-509.
89. Yu Z, Ju CH, Xu B, Wu JD, Jin LY (2010) Acupuncture for simple obesity: A systematic review of randomized controlled trials. Lishizhen Medicine and Materia Medica Research 21:434-436.
90. Wang ZL, Fu LX, Xiong J, Qi YZ, Li S (2010) Systematic Review of Therapeutic Effect on Acupuncture for Treatment of Urinary Incontinence after Stroke. Journal of Clinical Acupuncture and Moxibustion 26: 39-43.
91. Tang ZM, Zhang JM, Liu YR, Liu SY (2010) Acupuncture in the treatment of diabetic gastroparesis: a systemic review. China Tropical Medicine 10:235-238.
92. Li ZP, Yan XY (2010) Clinical Effect of Acupuncture for essential insomnia-A systematic review. Journal of Clinical Acupuncture and Moxibustion 26: 43-49.
93. Pang Y, Wu LB, Liu DH (2010) Acupuncture therapy for apoplectic aphasia: A systematic review. Chinese Acupuncture & Moxibustion 30: 612-616.
94. Mu JP, Liu L, Fang W, Cheng JM, Ao JB, et al (2010) Systematic review of needle-knife therapy for lumbar intervertebral disc protrusion. Chinese Journal of Information on Traditional Chinese Medicine 17:31-35.
95. Xiong J, Du YH, Liu JL, Lin XM, Sun P, et al (2010) Acupuncture versus western medicine for post stroke depression: A systematic review. The Journal of Evidence-Based Medicine 10:179-185,192.
96. Chen YW, Du YH, Xiong J, Sun P, Gao X, et al (2010) Acupuncture and moxibustion versus western medicine for benign prostatic hyperplasia: A systematic review. China Journal of Traditional Chinese Medicine and Pharmacy 25:902-906.
97. Zhao C,Mu JP,Cui YH,Yang L,Ma XP,et al (2010) Meta-analysis on acupuncture and moxibustion for irritable bowel syndrome. Chinese Archives of Traditional Chinese Medicine 28:961-963.
98. Xiao L, Chen YW, Du YH, Gao X, Lin XM, et al (2010) Systematic review of clinical randomized control trials of acupuncture for treatment of multiple tics-coprolalia syndrome. Lishizhen Medicine and Materia Medica Research 21:1199-1202.
99. Yuan SS, Zhang SY (2010) Meta-analysis of acupuncture for cognitive disorder after Stroke . Chinese Journal of Ethnomedicine and Ethnopharmacy 19:47-48.
100. Yang LH, Du YH, Xiong J, Liu JL, Wang YN, et al (2010) Acupuncture treatment for parkinson disease: A systematic review. Chinese Journal of Evidence-Based Medicine 10:711-717.
101. Yang F, Wang YL, Guo Q, Liu Y (2010) Systematic review of acupuncture for treatment of neruodermatitis in domestic clinical studies. Journal of Clinical Acupuncture and Moxibustion 26:1-7.
102. Yang ZB, Zhang WJ (2010) Systematic review of acupuncture treatment on eczema. International Journal of Triditional Chinese Medicine 32:250-251.
103. Yu L, Zhang Y, Chen C, Cui HF, Yan XK (2010) Meta-analysis on randomized controlled clinical trials of acupuncture for asthma. Chinese Acupuncture & Moxibustion 30:787-792.
104. Tang HL, Pang J, Lei LM, Gan W, Liu ZW (2010) Acupuncture for sub-health in clinical researches: A systematic review of clinical studies. Liaoning Journal of Traditional Chinese Medicine 37: 1188-1190.
105. Fan L, Fu WB, XU NG, Liu JH, Ou AH, et al (2010) Meta-analysis of randomized controlled trials on acupuncture and moxibustion treating depression. Chinese Journal of Gerontology 30:2561-2563
