# Supplementary material for: Risk of Bias Tool in Systematic Reviews/Meta-Analyses of Acupuncture in Chinese Journals
Source: PLoS One. 2011 Dec 9;6(12):e28130. doi: 10.1371/journal.pone.0028130 (PMC3235108; doi:10.1371/journal.pone.0028130)
Supplement: Text S3 — PRISMA Checklist. (DOC) [file pone.0028130.s003.doc]

| **Section/topic** | **#** | **Checklist item** | **Reported on page #** | **Brief description of how the criteria were handled in the meta-analysis** |
| --- | --- | --- | --- | --- |
| **TITLE** | | |  |  |
| Title | 1 | Identify the report as a systematic review, meta-analysis, or both. | - | The study was based on systematic reviews/meta-analysis. |
| **ABSTRACT** | | |  |  |
| Structured summary | 2 | Provide a structured summary including, as applicable: background; objectives; data sources; study eligibility criteria, participants, and interventions; study appraisal and synthesis methods; results; limitations; conclusions and implications of key findings; systematic review registration number. | √ | The abstract provided a structured summary including background, method, result and conclusion according to PLoS ONE Guidelines for Authors. |
| **INTRODUCTION** | | |  |  |
| Rationale | 3 | Describe the rationale for the review in the context of what is already known. | √ | Use of a risk of bias (ROB) tool has been encouraged and advocated to reviewers writing systematic reviews (SRs). Selective outcome reporting and other sources of bias are included in the Cochrane ROB tool. |
| Objectives | 4 | Provide an explicit statement of questions being addressed with reference to participants, interventions, comparisons, outcomes, and study design (PICOS). | - | Our objectives were to evaluate whether and to what extent the new Cochrane ROB tool has been used in Chinese journal papers of acupuncture. |
| **METHODS** | | |  |  |
| Protocol and registration | 5 | Indicate if a review protocol exists, if and where it can be accessed (e.g., Web address), and, if available, provide registration information including registration number. | √ | The protocol of this study was written in Chinese which wasn’t published. |
| Eligibility criteria | 6 | Specify study characteristics (e.g., PICOS, length of follow-up) and report characteristics (e.g., years considered, language, publication status) used as criteria for eligibility, giving rationale. | √ | Detailed inclusion and exclusion criteria were described in the methods section. |
| Information sources | 7 | Describe all information sources (e.g., databases with dates of coverage, contact with study authors to identify additional studies) in the search and date last searched. | √ | Five databases (CBM, TCM database, CJFD, CSJD and Wanfang Database) were systematically searched from inception to March 2011. |
| Search | 8 | Present full electronic search strategy for at least one database, including any limits used, such that it could be repeated. | √ | Text S1 |
| Study selection | 9 | State the process for selecting studies (i.e., screening, eligibility, included in systematic review, and, if applicable, included in the meta-analysis). | √ | Two reviewers independently screened and discussed. |
| Data collection process | 10 | Describe method of data extraction from reports (e.g., piloted forms, independently, in duplicate) and any processes for obtaining and confirming data from investigators. | √ | Data about general characteristics and “risk of bias” were independently extracted by two reviewers. |
| Data items | 11 | List and define all variables for which data were sought (e.g., PICOS, funding sources) and any assumptions and simplifications made. | √ | General information and the information related to risk of bias were extracted and analysed. Data was summarized using descriptive statistics (frequency, percentage). |
| Risk of bias in individual studies | 12 | Describe methods used for assessing risk of bias of individual studies (including specification of whether this was done at the study or outcome level), and how this information is to be used in any data synthesis. | - | We focused on whether and to what extent the ROB tool has been used in the included SRs/MAs. |
| Summary measures | 13 | State the principal summary measures (e.g., risk ratio, difference in means). | √ | We used descriptive statistics, such as frequency, percentage. |
| Synthesis of results | 14 | Describe the methods of handling data and combining results of studies, if done, including measures of consistency (e.g., I^2^) for each meta-analysis. | - | We didn’t combine results of studies. |

Page 1 of 2

| **Section/topic** | **#** | **Checklist item** | **Reported on page #** | **Brief description of how the criteria were handled in the meta-analysis** |
| --- | --- | --- | --- | --- |
| Risk of bias across studies | 15 | Specify any assessment of risk of bias that may affect the cumulative evidence (e.g., publication bias, selective reporting within studies). | - | Risk of bias across studies was not involved in this study. |
| Additional analyses | 16 | Describe methods of additional analyses (e.g., sensitivity or subgroup analyses, meta-regression), if done, indicating which were pre-specified. | - | Additional analyses (e.g. sensitivity or subgroup analyses) were not used in this study. |
| **RESULTS** | | |  |  |
| Study selection | 17 | Give numbers of studies screened, assessed for eligibility, and included in the review, with reasons for exclusions at each stage, ideally with a flow diagram. | √ | Figure 1 |
| Study characteristics | 18 | For each study, present characteristics for which data were extracted (e.g., study size, PICOS, follow-up period) and provide the citations. | √ | Table 1 |
| Risk of bias within studies | 19 | Present data on risk of bias of each study and, if available, any outcome level assessment (see item 12). | - | We focused on whether and to what extent the new Cochrane ROB tool has been used in SRs/MAs. |
| Results of individual studies | 20 | For all outcomes considered (benefits or harms), present, for each study: (a) simple summary data for each intervention group (b) effect estimates and confidence intervals, ideally with a forest plot. | - | We reported the result about using of risk of bias tool. |
| Synthesis of results | 21 | Present results of each meta-analysis done, including confidence intervals and measures of consistency. | - | Frequency and percentage were reported in this study. |
| Risk of bias across studies | 22 | Present results of any assessment of risk of bias across studies (see Item 15). | - | Risk of bias across studies was not involved in this study. |
| Additional analysis | 23 | Give results of additional analyses, if done (e.g., sensitivity or subgroup analyses, meta-regression [see Item 16]). | - | Additional analysis was not used in this study. |
| **DISCUSSION** | | |  |  |
| Summary of evidence | 24 | Summarize the main findings including the strength of evidence for each main outcome; consider their relevance to key groups (e.g., healthcare providers, users, and policy makers). | √ | We discussed the main findings including the use of the ROB tool in SRs/MAs. |
| Limitations | 25 | Discuss limitations at study and outcome level (e.g., risk of bias), and at review-level (e.g., incomplete retrieval of identified research, reporting bias). | √ | The four limitations were listed in the discussion section. |
| Conclusions | 26 | Provide a general interpretation of the results in the context of other evidence, and implications for future research. | √ | The Cochrane "risk of bias" tool has not been used in all SRs/MAs of acupuncture published in Chinese Journals after 2008. When the ROB tool was used, reporting of relevant information was often incomplete. |
| **FUNDING** | | |  |  |
| Funding | 27 | Describe sources of funding for the systematic review and other support (e.g., supply of data); role of funders for the systematic review. | √ | We described that “no current external funding sources for this study” in additional information. |

*From:*  Moher D, Liberati A, Tetzlaff J, Altman DG, The PRISMA Group (2009). Preferred Reporting Items for Systematic Reviews and Meta-Analyses: The PRISMA Statement. PLoS Med 6(6): e1000097. doi:10.1371/journal.pmed1000097

For more information, visit: **www.prisma-statement.org**.

Page 2 of 2

**Text S3 PRISMA Checklist.**

**Risk of Bias Tool in Systematic Reviews/Meta-analyses of Acupuncture in Chinese Journals**

Yali Liu ^1,2^, Shengping Yang ^1,3^, Junjie Dai ^1,3^, Yongteng Xu ^1,3^, Rui Zhang ^1,3^, Huaili Jiang ^1,3^, Xianxia Yan ^1,3^, Kehu Yang ^1,2^*

1 Evidence-Based Medicine Center, School of Basic Medical Sciences, Lanzhou University, Lanzhou, Gansu, China

2 Institute of Integrated Traditional Chinese and Western Medicine, Lanzhou University, Lanzhou, Gansu, China

3 The First Clinical Medical College of Lanzhou University, Lanzhou, Gansu, China

Corresponding Author:

Kehu Yang

Address: No. 199, Donggang West Road, Chengguan District, Lanzhou, Gansu, China, 730000

Phone: +86-931-8912767

E-mail: kehuyangebm2006@126.com
